# Patient-Related Metadata Reported in Sequencing Studies of SARS-CoV-2: Protocol for a Scoping Review and Bibliometric Analysis

**DOI:** 10.2196/58567

**Published:** 2025-04-22

**Authors:** Karen O'Connor, Davy Weissenbacher, Amir Elyaderani, Ebbing Lautenbach, Matthew Scotch, Graciela Gonzalez-Hernandez

**Affiliations:** 1 Department of Biostatistics, Epidemiology, and Informatics Perelman School of Medicine University of Pennsylvania Philadelphia, PA United States; 2 Department of Computational Biomedicine Cedars-Sinai Medical Center Los Angeles, CA United States; 3 Biodesign Center for Environmental Health Engineering Arizona State University Tempe, AZ United States; 4 Division of Infectious Diseases Department of Medicine University of Pennsylvania Philadelphia, PA United States; 5 Center for Clinical Epidemiology and Biostatistics Perelman School of Medicine University of Pennsylvania Philadelphia, PA United States; 6 College of Health Solutions Arizona State University Tempe, AZ United States

**Keywords:** SARS-CoV-2, COVID-19, genomic epidemiology, GISAID, GenBank, sequence records, patient-related metadata, scoping review, protocol

## Abstract

**Background:**

There has been an unprecedented effort to sequence the SARS-CoV-2 virus and examine its molecular evolution. This has been facilitated by the availability of publicly accessible databases, such as the GISAID (Global Initiative on Sharing All Influenza Data) and GenBank, which collectively hold millions of SARS-CoV-2 sequence records. Genomic epidemiology, however, seeks to go beyond phylogenetic (the study of evolutionary relationships among biological entities) analysis by linking genetic information to patient characteristics and disease outcomes, enabling a comprehensive understanding of transmission dynamics and disease impact. While these repositories include fields reflecting patient-related metadata for a given sequence, the inclusion of these demographic and clinical details is scarce. The current understanding of patient-related metadata in published sequencing studies and its quality remains unexplored.

**Objective:**

Our review aims to quantitatively assess the extent and quality of patient-reported metadata in papers reporting original whole genome sequencing of the SARS-CoV-2 virus and analyze publication patterns using bibliometric analysis. Finally, we will evaluate the efficacy and reliability of a machine learning classifier in accurately identifying relevant papers for inclusion in the scoping review.

**Methods:**

The National Institutes of Health’s LitCovid collection will be used for the automated classification of papers reporting having deposited SARS-CoV-2 sequences in public repositories, while an independent search will be conducted in MEDLINE and PubMed Central for validation. Data extraction will be conducted using Covidence (Veritas Health Innovation Ltd). The extracted data will be synthesized and summarized to quantify the availability of patient metadata in the published literature of SARS-CoV-2 sequencing studies. For the bibliometric analysis, relevant data points, such as author affiliations, citation metrics, author keywords, and Medical Subject Headings terms will be extracted.

**Results:**

This study is expected to be completed in early 2025. Our classification model has been developed and we have classified publications in LitCovid published through February 2023. As of September 2024, papers through August 2024 are being prepared for processing. Screening is underway for validated papers from the classifier. Direct literature searches and screening of the results began in October 2024. We will summarize and narratively describe our findings using tables, graphs, and charts where applicable.

**Conclusions:**

This scoping review will report findings on the extent and types of patient-related metadata reported in genomic viral sequencing studies of SARS-CoV-2, identify gaps in the reporting of patient metadata, and make recommendations for improving the quality and consistency of reporting in this area. The bibliometric analysis will uncover trends and patterns in the reporting of patient-related metadata, including differences in reporting based on study types or geographic regions. The insights gained from this study may help improve the quality and consistency of reporting patient metadata, enhancing the utility of sequence metadata and facilitating future research on infectious diseases.

**Trial Registration:**

OSF Registries osf.io/wrh95; https://doi.org/10.17605/OSF.IO/WRH95

**International Registered Report Identifier (IRRID):**

DERR1-10.2196/58567

## Introduction

### Background

Since the onset of the COVID-19 pandemic, there has been an unprecedented effort in genomic epidemiology (genomic epidemiology links pathogen genomes with associated metadata to understand disease transmission) to sequence the virus, study its transmission, and examine molecular evolution. Public repositories, such as the GISAID (Global Initiative on Sharing Avian Influenza Data) [1] and the National Center for Biotechnology Information (NCBI)’s GenBank [2] host millions of SARS-CoV-2 sequence records. As of September 2024, GISAID contains 16.9 million sequences, while over 8.9 million have been deposited in GenBank.

The availability of this vast amount of genomic data has facilitated significant discoveries, particularly in phylogenetic (the study of evolutionary relationships among biological entities) and phylodynamic (the reconstruction of epidemiological and immunological processes from the shape of phylogenetic tree relating infections) studies [3-5]. Beyond phylogenetic studies, genomic epidemiology aims to understand the transmission dynamics, evolution, and impact of infectious diseases by analyzing the genetic information of pathogens and linking it to patient demographics and disease outcomes [6,7]. This work enables the tracking of the spread of pathogens, identifying high-risk populations, and discovering genetic factors that influence disease transmission, severity, and treatment response [6,8]. This knowledge can, in turn, inform public health strategies, guide the development of targeted interventions, and improve the overall understanding of infectious diseases [9].

Ideally, patient geographic, demographic, and clinical information (such as disease severity and outcome) would be included in the sequence metadata upon its submission to the repository. Both GISAID and GenBank frequently provide the location of the infected host information in their sequence metadata, however, the reported location granularity may vary and often lacks important details such as patient travel history. Similarly, patient demographic and clinical information is rarely complete. A review of available metadata in these 2 large public repositories for SARS-CoV-2 sequences, conducted by the authors in April 2023, found 58.34% (8,943,721/15,329,810) of sequences in GISAID do not include the specific age and 58.58% (8,980,046/15,329,810) do not include the specific gender of the infected host. The information for these may be entered as unknown (eg, “not available,” “declined,” “not reported”). GenBank lacks standardized fields to include age or gender information with sequence submissions.

Several studies have highlighted the importance and challenges of metadata reporting in SARS-CoV-2 research and identified several shortcomings in the metadata that accompany these sequences [10,11], particularly deficiencies in the completeness and standardization of the reported data. Proposals have been made for the standardization of this data, but they have not been widely adopted [12]. Another review highlighted the importance of patient-related metadata for genomic epidemiology in general but provided no assessment of the availability of these data [13]. These studies collectively emphasize the critical need for improved metadata reporting practices, but they do not provide a comprehensive analysis of patient-related metadata reporting specifically in SARS-CoV-2 sequencing studies across multiple repositories or publications such as what we propose.

Previous research has found that sequence metadata can be enhanced for the location of the infected host using natural language processing and machine learning methods to automatically extract and link this information to the sequence record [14,15]. This patient-related information, or at least a subset of it, may be reported in the published studies of those who obtained and performed the genomic sequencing allowing these methods to be extended and applied to SARS-CoV-2 sequences. However, the extent to which patient-related geographic information, such as their residence or travel history, is reported in SARS-CoV-2 sequencing studies remains largely unexplored. Similarly for patient demographics or other clinical information. Our review aims to bridge this gap in understanding by quantifying the extent and types of patient-related metadata reported in published genomic viral sequencing studies of SARS-CoV-2.

Traditionally, identifying studies for a review requires the development of a detailed search strategy of databases using keywords and index terms, querying the titles and abstracts of published papers. The selection of keywords greatly influences search results, leading to potentially missed studies and the inclusion of potentially irrelevant studies. Moreover, for the particular focus of our study, discussions of sequencing are often confined to the methods section of papers, rendering title and abstract screening less informative. While more than 437,000 research papers [16] related to SARS-CoV-2 and the pandemic have been published, there is sparse linkage between the sequence and publication databases. This makes it difficult to identify publications relevant to the sequences, and severely limits meta-analyses and scaling studies by using datasets produced by different investigators. To overcome these limitations, we propose using an automated classifier to identify relevant studies for review. In addition, we will use a traditional database search to validate and compare the approaches.

A bibliometric analysis uses different methods and data points to quantify the trends and assess the impact of publications in a specific field [17]. While several bibliometric analyses have investigated COVID-19–related research trends in general [18-20] and in specific fields such as neurology [21], long COVID [22], and medical imaging [23], or for specific geographic locations such as Africa [24], no bibliometric analysis exists specifically focused on reporting patterns of patient metadata in sequencing studies related to the SARS-CoV-2 genome, nor examined how reporting practices evolved throughout the pandemic. We hypothesize that using bibliometric indicators, differences in metadata reporting will be seen based on study type, institution, and size, with smaller, clinical-based studies reporting more information than larger, surveillance studies.

Our aims with this review and analysis are to address the gaps in the understanding of the extent and quantity of patient-related metadata reporting in genomic sequencing studies by providing a comprehensive assessment of this reporting in the published SARS-CoV-2 sequencing studies. Using bibliometric methods, we will systematically examine factors that may influence metadata reporting in publications associated with SARS-CoV-2 sequence reporting over the course of the pandemic. By combining detailed content analysis of patient metadata with bibliometric analysis, we can identify factors that influence reporting practices, such as journal or institutional policies, international collaborations, or study types as well as highlight the gaps in reporting that may hinder the advancement of genomic epidemiology studies of the COVID-19 pandemic.

### Primary Research Objectives

The primary research objectives are the following: (1) To quantitatively assess the extent and quality of patient-reported metadata, including demographic, clinical, and geographic information, in papers reporting original whole genome sequencing of the SARS-CoV-2 virus. (2) To perform a comprehensive bibliometric analysis to ascertain differences and discernible patterns between papers that include patient metadata and those that do not, thereby providing insights into the characteristics and factors associated with the reporting of patient data in the literature. (3) To evaluate the efficacy and reliability of a machine learning classifier in accurately identifying relevant papers for inclusion in the scoping review, enhancing the efficiency and effectiveness of this study’s selection process.

## Methods

### Study Design

Our scoping review will follow the methodological framework identified by Arksey and O’Malley [25] and will be reported in line with the PRISMA-ScR (Preferred Reporting Items for Systematic Reviews and Meta-Analyses extension for Scoping Reviews) checklist [26] (Multimedia Appendix 1).

### Data Sources

We will use the National Institutes of Health’s LitCovid collection [16] for our machine learning classification. LitCovid is a curated collection of scholarly papers related to COVID-19. As of November 2024, the collection contains over 437,000 publications from 8000 journals and is updated daily. LitCovid includes published papers as well as preprints. Additionally, we will independently search Ovid MEDLINE and PubMed Central directly using a 2-faceted search strategy and the NCBI e-utilities program to find publications linked to sequences. This combined approach will help ensure a comprehensive coverage of the literature for our study.

### Search Strategy

#### Classification Model

The details of our classification model have been previously reported [27]. Briefly, our classification model was trained using manually annotated data. A full-text search strategy was developed to filter the LitCovid collection resulting in a corpus of targeted papers for annotation. The papers identified through the pipeline were annotated by 2 experienced annotators using the INCEpTION annotation tool [28] and following methodically created annotation guidelines. The annotators reviewed the full text of 245 randomly selected papers and labeled sentences, which confirmed this study’s performance of SAR-CoV-2 sample sequencing from human specimens. The interannotator agreement for the annotation was measured using Cohen κ. The score for agreement on whether the paper reported original viral sequencing was 1, and sentence agreement, which was calculated on papers that reported sequencing (n=74), was moderate [29] (k=0.71). Disagreements were resolved by a third annotator. The final annotated corpus consisted of 50,918 sentences from 245 papers. There were 74 papers that reported SARS-CoV-2 sequencing and, within these papers, 347 sentences were annotated as positive. We split our annotated dataset into 3 random sets: a training set of 147 papers (31,885 sentences), a validation set of 49 papers (9017 sentences), and a test set of 49 papers (10,016 sentences). For our classifier, we pretrained a transformer-based neural network, specifically a bert-base-uncased [30] model from the Hugging Face library. On the held-out test set, the classifier achieved an *F*_1_-score of 0.48 (precision=0.492 and recall=0.469) for identifying sentences that provided evidence of generating new SARS-CoV-2 sequences. While the classifier achieved moderate performance at the sentence level, assessing the performance at the paper level, meaning at least 1 sentence in the paper that indicated sequencing was detected, the classifier achieved a more robust performance of *F*_1_-score of 0.8 (precision=0.667 and recall=1).

#### Database Search Strategy

To evaluate our classifier and identify studies that may have been missed due to classification errors or the lack of full text in the LitCovid collection, we will create a search strategy to independently search MEDLINE and PubMed Central. We will develop a 2-faceted search strategy to find “SARS-CoV-2” and “whole genome sequencing” related publications. We will use the search strategy developed for the LitCovid collection with additional keywords added to identify studies that report whole genome sequencing. A sample search strategy is found in Multimedia Appendix 2. Additionally, we will search for publications linked to SARS-CoV-2 sequences using the NCBI’s e-utilities eLink programming application programming interface. We will also search gray literature sources, such as Google Scholar and review the reference lists of included studies [31].

A publication date restriction of December 2019 onward will be used in the searches as this review is focused on SARS-CoV-2 sequencing studies. No language restrictions will be placed on the searches, although financial and logistical restraints will not allow translation from all languages.

#### Inclusion or Exclusion Criteria

Papers positively identified by our classifier and our search results will be reviewed for inclusion in the review based on the criteria outlined in Table 1.

**Table 1:** Inclusion and exclusion criteria for the scoping review.

Facet	Inclusion criteria	Exclusion criteria
Sample origin	Individual human subject	Nonhuman sources (eg, mice, bats, and ferrets) Wastewater Microbiome Cloned or cell culture virus
Sequencing type	Whole genomic sequencing, including partial or complete sequence results	Studies will be excluded if the following sequencing methods were exclusively performed: Polymerase chain reaction or loop-mediated isothermal amplification for viral detection Single-cell sequencing Gene expression studies Protocol validation studies on cell culture virus Exome sequencing
Study design	Any type of peer-reviewed or preprint study reporting on the original sequencing of SARS-CoV-2 samples. The study reports the deposit of the sequences into a data repository	Any other study design Any study that does not report the depositing of sequences into a data repository
Publication dates	December 2019 or later	Before December 2019
Language	All	None

#### Screening and Paper Selection

**Figure 1:**
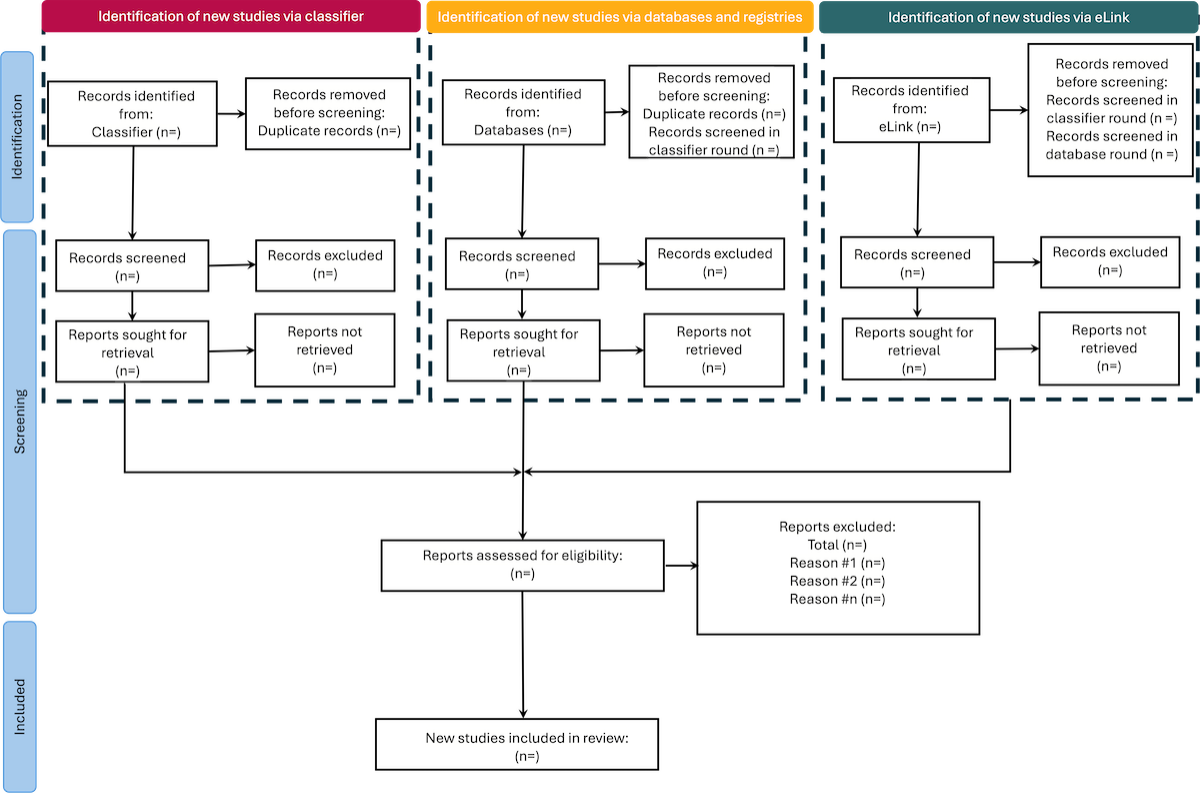
Flow diagram of proposed screening of identified papers. We will first screen papers from our classifier, then we will screen those identified from database searches to ensure there are no duplicate records screened.

Two reviewers will perform title and abstract screening using the Covidence systematic review management tool with any disagreements resolved by discussion. We will screen the papers from the different methods in a systematic order (Figure 1). First, we will validate and screen the results from our classifier’s predictions on the LitCovid collection. Next, we will screen the papers obtained from our database searches. All results will be uploaded to a Zotero library where duplicate results will be removed. We will then identify if a paper is in the LitCovid collection; those that are not will be moved to screening. For those that are, we will assess whether the paper was screened in the first round, those that were not will be screened in this round. Lastly, for papers identified as having links to GenBank records through NCBI’s eLink programming application programming interface, we will identify if any of the resulting papers had been screened in the previous 2 rounds, those that have not will then be screened.

Two independent reviewers will also conduct a full-text review in Covidence. To ensure interrater reliability, a subset of 10% of the screened studies will be independently reviewed by both reviewers. We will assess the level of agreement between reviewers using the Cohen κ coefficient [29]. Any discrepancies will be resolved through discussion. We will report the excluded studies with the reason for exclusion.

### Data Extraction

Data extraction will be conducted in Covidence. The reviewers will examine the full text of the papers, including any supplementary files, for data extraction. The customizable interface will be designed to prompt the reviewer to extract various details, such as general publication information, study characteristics, sequencing specifics, and the presence or absence of the patient’s demographic, clinical, or geographic information about where the patient resides or had traveled before sample collection, or the location of where the sample was collected. For studies with reported patient metadata, we will note whether information is reported per individual or in aggregate. For missing or incomplete metadata, we will categorize the absence using the following classifications: explicitly withheld for privacy, deidentified before sequencing, partially reported, or not reported. Furthermore, the section where the reported patient metadata within the papers was reported will be noted, for example, text, table, or supplemental materials. An example of the data extraction form can be found in Table 2. As this scoping review aims to report on the current state of published reports of patient-related metadata, we will not contact authors for any missing or additional data not found in the paper.

**Table 2:** Example of data that will be extracted from included studies.

Prompt			Response
**Publication information**			
	Study name	Free text	
	Paper title	Free text	
	Year of publication	YYYY	
	Publication type	Journal, conference, and preprint	
**Study and sequence information**			
	Study objective	Free text	
	Location of study (country)	Free text	
	Number of patients	Free text	
	Number of samples sequenced	Free text	
	Short description of how the generated sequences were used in the paper	Free text	
	Repository sequences deposited to	GISAID^a^, GenBank, other, or NR^b^	
	For studies with >1 patient, are sequences linked to a patient?	Yes or no	
**Patient demographic information reported**			
	Age	Yes or no	
	Gender	Yes or no	
	Race or ethnicity	Yes or no	
	If yes to any of the above, where in the paper was the information located	Text, table, or supplemental	
	Reporting level	Individual or aggregate	
	If not reported, the reason	Privacy, deidentified, NR, or partial	
**Patient clinical** **information reported**			
	Symptoms	Yes or no	
	Severity	Yes or no	
	Inpatient or outpatient	Yes or no	
	Treatments	Yes or no	
	Outcomes	Yes or no	
	If yes to any of the above, where in the paper was the information located	Text, table, and supplemental	
	Reporting level	Individual or aggregate	
	If not reported, the reason	Privacy, deidentified, NR, or partial	
**Patient geographic** **information reported**			
	Location of residence	Yes or no	
	Travel information	Yes or no	
	If yes to any of the above, where in the paper was the information located	Text, table, or supplemental	
	Reporting level	Individual or aggregate	
	If not reported, the reason	Privacy, deidentified, NR, or partial	

^a^GISAID: Global Initiative on Sharing All Influenza Data.

^b^Not reported.

We will test the initial extraction form on a subset of papers and revise it as needed.

For bibliometric analysis, all pertinent data points will be extracted for studies included in our review including, author location and institution information, journal, study type, citation metrics, and author keywords or Medical Subject Headings terms when available.

### Data Analysis

The extracted data will be synthesized and summarized to quantify the availability of patient metadata in the published literature of SARS-CoV-2 sequencing studies using an exported spreadsheet from Covidence. We will summarize and narratively describe our findings, using tables, graphs, and charts when applicable, regarding the number of sequences covered in our included studies, the distribution of the sequences in the respective repositories, and the quantity and type of reported patient metadata in the papers.

For the bibliometric analysis, data will be analyzed and visualized using the VOSviewer software or the *bibliometrix* [32] package for R (R Foundation). These will include publication metrics (eg, annual trends, and distribution by journal and country), author metrics (eg, collaboration networks or productivity), and citation analysis (eg, total and average citations, or highly cited papers). We will present the geographical location of the paper’s authors using maps to show the geographic distribution of research output and report our findings, including the most frequent journals and paper types using narrative descriptions or tables. We will use the data extracted from our review to analyze differences between studies that reported patient metadata from those that did not. Co-occurrence networks of author keywords will be presented to highlight the frequency and differences in themes and study type (eg, clinical study, case report, and surveillance study) between these reporting groups. We will analyze coauthorship networks and institutional collaborations to assess if highly collaborative studies are associated with more comprehensive metadata reporting. We will also analyze associations between study location, the potential impact of journal-related policies or characteristics, and the extent of metadata reporting. Specifically, we will examine the proportion of studies reporting different types of metadata (demographic, clinical, and geographic), trends in metadata reporting over time, and potential correlations between metadata reporting and other bibliometric indicators such as citations or journal impact factors. In addition to VOSviewer and *bibliometrix*, we will use the R statistical software to develop scripts for specific analyses related to metadata reporting trends.

As this is a scoping review (and not a systematic review), accepted practice [33] indicates that it need not include an assessment of the methodological quality (risk of bias assessment) of the papers or conduct any evidence synthesis.

### Ethical Considerations

This scoping review will consist of collecting and reviewing publicly available data from previously published studies and does not require any ethical approval. Furthermore, quantitative results will be reported in aggregate across the included studies. The results and findings of the completed scoping review will be disseminated through the submission of a paper for peer-reviewed publication and through scientific conferences. This paper will reference this protocol, and any changes or deviations made from this protocol will be acknowledged and justified.

## Results

This protocol has been registered at the Open Science Framework registries. This study is expected to be completed in early 2025. Our classification model has been developed and we have classified publications in LitCovid published through February 2023. As of September 2024, papers through August 2024 are being prepared for processing. Screening is underway for validated papers from the classifier. Direct literature searches and screening of the results began in November 2024. We will quantitatively summarize and narratively describe our findings, using tables, graphs, and charts when applicable.

## Discussion

### Principal Findings

The anticipated findings of this scoping review will provide a comprehensive overview of the current state of patient-related metadata reporting in SARS-CoV-2 sequencing studies. We expect to identify gaps in reporting practices, variations across different types of studies or geographic regions, and potential areas for improvement in metadata reporting standardization. In addition to the findings of our scoping review, the bibliometric analysis will likely identify several other important trends and patterns in the reporting of patient-related metadata. For example, the analysis may find that the reporting of patient-related metadata is more common in certain types of studies, or that it is more likely to be reported in studies from certain geographic regions. The findings of the scoping review and bibliometric analysis will provide valuable insights into the factors that influence the reporting of patient-related metadata and will help to inform future research on this topic.

The COVID-19 pandemic has spurred an unprecedented volume of research, including extensive efforts in genomic sequencing of SARS-CoV-2. However, the utility of these sequences for genomic epidemiology may not be fully realized due to the unavailability of relevant metadata about the patient from whom the specimen was obtained [34]. Shortcomings of this metadata that may accompany these sequences in the data repositories have been extensively noted [10-12]. Methods exist that facilitate the extraction of this data from other resources, such as published literature [14,15,35]. The identification and quantification of the metadata in literature may aid in advancing future research.

### Future Directions

Our study may lay the groundwork for determining the feasibility of the development of automated methods to extract patient-related metadata from publications to enrich sequences. These enriched sequences can be made available through a publicly shared repository. The availability of such a comprehensive resource could facilitate studies that compare how the inclusion of additional metadata impacts the conclusions and utility of genomic epidemiology studies. This could help quantify the importance of comprehensive metadata reporting, and potentially provide the impetus for researchers to improve their reporting practice.

Beyond the practices of researchers, there may be other factors that determine whether the patient metadata is published, such as journal data-sharing policies. Based on the findings of this scoping review researchers could develop and propose standardized guidelines for reporting patient-related metadata in SARS-CoV-2 sequencing studies. These guidelines could help improve the consistency and completeness of metadata reporting across future studies, enhancing the value of genomic sequences for epidemiological research.

Moreover, our study may reveal insights into the role privacy concerns play in the reporting of relevant patient metadata. This insight could guide targeted interventions to improve reporting practices while also addressing critical patient privacy concerns. Future work could explore the development of privacy-preserving methods for sharing more comprehensive metadata.

By providing a comprehensive overview of current metadata reporting practices, the results of this scoping review may support efforts to enhance both the completeness and ethical handling of patient-related metadata in genomic epidemiology research. These improvements could significantly advance our understanding of SARS-CoV-2 transmission dynamics and inform strategies for managing this and future pandemics.

### Strengths and Limitations

We propose a novel approach to identify relevant papers with the development of an automated classifier that will locate within the text of the paper sentences that indicate viral genome sequencing was performed in the paper. This method necessitates openly available, machine-readable texts which could bias our sample from this search to information in open-access papers. This bias should be limited in this study, however, as there was a commitment from publishers early in the COVID-19 pandemic to make content related to the pandemic open and available [36]. Furthermore, we will also conduct an independent search from databases outside of LitCovid to identify any potentially missed papers from our classifier or gaps in the LitCovid collection ensuring a more comprehensive and relevant collection of papers to include in our review. Still, there remains the possibility that some relevant studies may be missed due to search limitations which may lead to an under or overestimation of the extent of metadata reporting. While we aim to follow the best practices in methodology and reporting by adhering to the PRISMA-ScR checklist, we do deviate from standard practice for identifying studies through the use of a classifier. This approach will allow us to identify sequencing studies that may not be apparent from traditional title or abstract screening alone. Other limitations exist, such as potential limitations in reported patient metadata [37,38] and the focus on SARS-CoV-2 sequencing studies, which may limit the applicability of our findings to other pathogens or pandemics. There may also be a gap in publication time between the depositing of sequences and the publication of the paper. Furthermore, reporting patterns may differ from early in the pandemic due to the urgent need to disseminate information, reporting practices and requirements in publications may have changed over the course of the pandemic, and research priorities may have changed as the pandemic continued. Any of these scenarios may affect the ability to draw definitive conclusions about trends in metadata reporting over time.

### Conclusion

This protocol outlines the steps that we will take in our scoping review which will be supported by an automated classifier and bibliometric analysis. We will fill the knowledge gap regarding the extent and types of patient-related metadata reported in genomic viral sequencing studies of SARS-CoV-2 and will provide valuable insights by identifying themes and trends in the published literature. The results of this study may encourage improved and standardized reporting practices which will significantly enhance the utility of sequence metadata and aid in advancing our understanding of the SARS-CoV-2 or any future pandemic. Future research can build upon our study to address these gaps and enhance reporting practices in this field.

## Appendix

Multimedia Appendix 1 PRISMA-ScR (Preferred Reporting Items for Systematic Reviews and Meta-Analyses extension for Scoping Reviews) checklist.

Multimedia Appendix 2 Sample search strategy for Ovid MEDLINE.

## Abbreviations

GISAID: Global Initiative on Sharing All Influenza Data
NCBI: National Center for Biotechnology Information
PRISMA-ScR: Preferred Reporting Items for Systematic Reviews and Meta-Analyses Extension for Scoping Reviews

## Data Availability

This study will analyze and synthesize previously published information. Data-sharing does not apply to this paper as no datasets were generated or analyzed during this study. We will submit for publication the completed scoping review and bibliometric analysis. At that time, any extracted data and data generated in our analysis will be made available with the publication.
