# Supplementary material for: Patient-Related Metadata Reported in Sequencing Studies of SARS-CoV-2: Protocol for a Scoping Review and Bibliometric Analysis
Source: JMIR Res Protoc. 2025 Apr 22;14:e58567. doi: 10.2196/58567 (PMC12056431; doi:10.2196/58567)
Supplement: Multimedia Appendix 2 [file resprot_v14i1e58567_app2.docx]

| 1 | exp Coronavirus Infections/ | 288344 |
| --- | --- | --- |
| 2 | exp Coronavirus/ | 194321 |
| 3 | (coronavirus* or corona virus* or OC43 or NL63 or 229E or HKU1 or HCoV* or ncov* or covid* or sars-cov* or sarscov* or Sars-coronavirus* or Severe Acute Respiratory Syndrome Coronavirus* or "Kawasaki like paediatric inflammatory multisystem syndrome" or "Kawasaki like pediatric inflammatory multisystem syndrome" or "PIMS-TS" or "Kawa-COVID-19" or "MIS-C" or "multisystem inflammatory syndrome in children" or pediatric multisystem inflammatory disease).mp. | 464200 |
| 4 | (or/1-3) and ((20191* or 202*).dp. or 20190101:20301231.(ep).) | 450785 |
| 5 | 4 not (SARS or SARS-CoV or MERS or MERS-CoV or Middle East respiratory syndrome or camel* or dromedar* or equine or coronary or coronal or covidence* or covidien or influenza virus or HIV or bovine or calves or TGEV or feline or porcine or BCoV or PED or PEDV or PDCoV or FIPV or FCoV or SADS-CoV or canine or CCov or zoonotic or avian influenza or H1N1 or H5N1 or H5N6 or IBV or murine corona*).mp. | 200757 |
| 6 | ((pneumonia or covid* or coronavirus* or corona virus* or ncov* or 2019-ncov or sars*).mp. or exp pneumonia/) and Wuhan.mp. | 8860 |
| 7 | (2019-ncov or ncov19 or ncov-19 or 2019-novel CoV or sars-cov2 or sars-cov-2 or sarscov2 or sarscov-2 or SARS-2-nCoV or SARS-2-Cov or SARS-COV-19 or Sars-coronavirus2 or Sars-coronavirus-2 or SARS 2 coronavirus* or Severe Acute Respiratory Syndrome-CoV-2 or SARS-like coronavirus* or coronavirus-19 or covid19 or covid-19 or covid 2019 or ((novel or new or nouveau) adj2 (CoV or nCoV or covid or coronavirus* or corona virus or Pandemi*2)) or ((covid or covid19 or covid-19 or SARS-CoV-2) and pandemic*2) or (coronavirus* and pneumonia)).mp. | 442262 |
| 8 | (COVID-19 or SARS-CoV-2).rx,px,ox,rn. or (COVID-19 or COVID-19 serotherapy or ORF7b protein, SARS-CoV-2 or ORF6 protein, SARS-CoV-2 or ORF8 protein, SARS-CoV-2 or pediatric multisystem inflammatory disease, COVID-19 related or envelope protein, SARS-CoV-2 or ORF7a protein, SARS-CoV-2 or spike protein, SARS-CoV-2 or ORF3a protein, SARS-CoV-2 or COVID-19 drug treatment or severe acute respiratory syndrome coronavirus 2 or membrane protein, SARS-CoV-2 or ORF1ab polyprotein, SARS-CoV-2 or nucleocapsid protein, Coronavirus or COVID-19 vaccine or COVID-19 diagnostic testing).os,ps,rn,rs. | 39881 |
| 9 | 5 or 6 or 7 or 8 | 448727 |
| 10 | exp Genome, Viral/ | 67382 |
| 11 | (genome* or genomic* or genotype* or sequenc*).mp. | 2873817 |
| 12 | exp High-Throughput Nucleotide Sequencing/ | 56334 |
| 13 | (next generation sequenc* or high-throughput sequenc* or deep sequenc* or whole genome sequenc*).mp. | 139248 |
| 14 | 10 or 11 or 12 or 13 | 2886707 |
| 15 | 9 and 14 | 20218 |
| 16 | 15 and 20191201:20301231.(dt). | 19660 |
